# Research and Remedies

**Published:** 1914-01-31

**Authors:** 


### RESEARCH AND REMEDIES.

### X-Rays and Kidney Diseases.

During the last year or two surgical specialists in kidney diseases have taken into favour a particular method of examination which is found to be frequently of great assistance in diagnosis. This is the method of filling the ureters and pelvis of the kidney with a solution of a silver product known as collargol, which is opaque to $x$-rays, and of then taking an $x$-ray photograph of the abdomen before the collargol is allowed to flow out of the urinary system. The famous Mayo brothers, of Rochester, Minn., U.S.A., have specially devoted themselves to perfecting this means of diagnosis, which has now been a good deal used by urologists in this country also. The photographs thus obtained give a picture of the pelvis of the kidney and of the ureter; and the accumulating experience shows that, as in other $x$-ray diagnosis, the interpretation of the plates is not always simple and straightforward.

Childs and Spitzer, in the *Journal of the American Medical Association*, have thus examined a great many patients whose kidneys were normal; and their findings are of interest because the study of the abnormal is, or ought to be, based on a previous thorough study of the normal. They conclude that pyelography by $x$-rays is not only justifiable, but essential as a diagnostic measure. That the lower border of the renal pelvis is concave. That the pelvis of the kidney does not normally hold more than 15cc., and one which holds more is probably dilated. That angulations, curves, and kinks of the ureter are found in normal persons. That pain, shock, and urinary fever after pyelography are due to rapid distension of the pelvis rather than to any special drug used. They can be avoided, as a rule, by great care in introducing the opaque substance, though when the pelvis is completely filled they may be unavoidable. That the normal ureter varies considerably in size and shape: the variations produced by peristalsis also complicate the interpretation of the radiographic pictures.

### The Treatment of Burns.

For a long time surgeons have been dissatisfied with the old methods of treating burns, and have paid much attention to lines of treatment which shall minimise the sepsis which so often endangers life and health after the initial shock of a bad burn has been recovered from. Picric acid has had a long vogue, and is certainly vastly better than the old-fashioned carron oil, so beloved of past generations; but it has disadvantages, and its use does not abolish, though it does limit, sepsis. In the *Therapeutic Gazette*, Stoops, of Texas, upholds the use of liquor cresolis comp. (U.S.P.), first suggested five years ago by Tennant. His routine is as follows: Bathe the affected surface with warm 1 per cent. solution of liquor cresolis until débris is removed and the parts are anæsthetic. Puncture blebs and express serum. Apply strips of gauze or of paraffin paper smeared with vaseline containing 1 per cent. of liq. cresolis co. Cover with cotton, and bandage. This dressing is allowed to remain on four or five days, after which it is renewed. It is said that toxic effects are much more uncommon than with picric acid; that the application causes no pain, even when first applied; and that it does not coagulate albumin in the tissues or in the fluid of the bulla. The method seems on this showing to be at least worthy of an independent and impartial trial.

